# Skeletal site-specific variations in myeloid cells: insights from single-cell RNA sequencing of the mandible and femur

**DOI:** 10.1093/jbmrpl/ziaf074

**Published:** 2025-04-24

**Authors:** Rachel Clark, Ryan Keesler, Adnan Ali, Marissa Macchietto, Sarah A Munro, Rachel Uppgaard, Elizabeth Bradley, Amy Tasca, Kim Mansky

**Affiliations:** Oral Biology Graduate Program, University of Minnesota School of Dentistry, Minneapolis, MN 55455, United States; Division of Orthodontics, Department of Developmental and Surgical Sciences, University of Minnesota School of Dentistry, Minneapolis, MN 55455, United States; Department of Biochemistry, Genetics and Biophysics, University of Minnesota, Minneapolis, MN 55455, United States; Minnesota Supercomputing Institute, University of Minnesota, Minneapolis, MN 55455, United States; Minnesota Supercomputing Institute, University of Minnesota, Minneapolis, MN 55455, United States; Division of Oral Surgery, Department of Developmental and Surgical Sciences, University of Minnesota School of Dentistry, Minneapolis, MN 55455, United States; Department of Orthopedic Surgery and Stem Cell Institute, University of Minnesota, Minneapolis, MN 55455, United States; Division of Orthodontics, Department of Developmental and Surgical Sciences, University of Minnesota School of Dentistry, Minneapolis, MN 55455, United States; Division of Orthodontics, Department of Developmental and Surgical Sciences, University of Minnesota School of Dentistry, Minneapolis, MN 55455, United States

**Keywords:** inflammation, transcription, osteoclasts, craniofacial, sequencing

## Abstract

To understand differences that exist between the cell populations in different skeletal sites, we performed an unbiased genetic survey via single-cell RNA sequencing of CD11b+ myeloid cells from mandibular- and femur-derived bone marrow of 2-mo-old C57BL/6 mice. Our results reveal transcriptomic evidence that suggests a uniquely inflammatory genetic profile of the mandibular-derived CD11b+ cells. The monocyte cell population found within the CD11b+ cells analyzed by scRNA-seq expressed *Csf1r* and *Tnfrsf11a* suggesting that this population contained osteoclast precursors. Osteoclasts of the craniofacial region facilitate processes such as tooth eruption and jawbone development. Evidence from multiple researchers suggests that craniofacial osteoclasts exhibit differences in size, gene expression, and activity compared to their counterparts within the appendicular skeleton. A biological mechanism to explain the observable difference between craniofacial osteoclasts and osteoclasts in the long bones has not been previously explored. This monocyte population had enhanced inflammatory gene expression by qRT-PCR which correlated with an increase in select areas of open chromatin by assay for transposase-accessible chromatin using sequencing. Further exploration into a specific upregulated gene determined KLF4 was both necessary and important for proper differentiation in mandibular- but not femur-derived cells.

## Introduction

Osteoclasts are large, multinucleated cells derived from the myeloid lineage that are responsible for bone resorption throughout the body. Although an osteoclast’s primary function is bone resorption, there is evidence of resorption performed by these cells, facilitating site-specific processes throughout the body. One example where osteoclasts perform site-specific processes is the craniofacial region, facilitating events such as tooth eruption and jawbone development.

Previous studies from multiple researchers suggest the size, resorptive abilities, and gene expression of osteoclasts derived from the marrow of the mandible differ compared to osteoclasts isolated from the marrow of the femur.^1–5^ While these studies suggest cells from the mandible have unique basal and induced osteoclastic potential, these studies come to different conclusions on what those differences truly are. We have previously shown that monocytes derived from the mandibular bone marrow space cultured in the presence of macrophage colony-stimulating factor 1 (M-CSF) and RANKL form larger osteoclasts, with a corresponding increase in expression of key osteoclastogenic regulatory genes.^6^ However, a potential biological mechanism to explain why these site-specific differences exist between osteoclast populations in the craniofacial region as compared to these populations in the appendicular skeleton has not been investigated. By exploring, in an unbiased manner, these differences using transcriptomic profiling of myeloid cells, this study aims to advance our knowledge toward understanding the biological differences between site-specific myeloid-derived cells, which have the potential to become osteoclast precursors.

With the advent of single-cell RNA sequencing (scRNA-seq) technology, the ability to explore the heterogeneity occurring in cell types and cell populations between different skeletal sites and in different pathological conditions now exists. Recent scRNA-seq studies comparing the complete bone marrow cells of the mandible and femur reveal differences between these cell populations in these 2 skeletal sites.^7^^,^^8^ Both studies revealed a higher proportion of mature immune cells, suggesting a need to maintain immune homeostasis in the craniofacial bones in the presence of oral microorganisms.^7^^,^^8^ Additionally, analysis revealed the myeloid cell population in the mandible has a different transcriptome signature compared to femur-derived myeloid cells.^7^

In this study, we analyze the differences in transcriptomic profiles of CD11b+ myeloid cells within the mandibular- and femoral-derived bone marrow using scRNA seq. We explore chromatin accessibility differences of mandibular- and femur-derived monocytes using the assay for transposase-accessible chromatin with sequencing (ATAC-seq). Our overall aim is to develop a refined transcriptomic understanding of factors driving the previously observed differences between myeloid cell populations of these 2 distinct skeletal sites.

Using these techniques, our study uncovers a distinct inflammatory transcriptomic profile of the mandibular-derived CD11b+ cells as compared to the femur-derived cells by scRNA-seq. Additionally, we show increased expression of key inflammatory genes in both mouse and human mandibular-derived monocytes as compared to cells derived from the femur, with a correlating increase in open chromatin regions. Using our transcriptomic profiling, Krüppel-like factor 4 (KLF4), a zinc-finger containing transcription factor and an upstream regulator of key metabolic transporters, was necessary for mandibular but not femur-derived osteoclast differentiation.

## Materials and methods

### Ethics

The use and care of mice used in this study were reviewed and approved by the University of Minnesota (UMN) Institutional Animal Care and Use Committee, IACUC protocol number 2402-41820A. Euthanasia was performed by CO_2_ inhalation. This study complies with the updated ARRIVE guidelines. The collection of human samples in this study was reviewed and approved by the UMN Institutional Review Board protocol number STUDY00017926.

### Single-cell RNA sequencing preparation

Primary bone marrow macrophages (BMMs) were harvested from the femurs of 2-mo-old male and female C57Bl/6J mice. Femurs were dissected, and adherent tissue was removed. The ends of the bone were cut, and the marrow was flushed from the inner compartments. Mandibles were dissected into 2 portions, a right and left side, and adherent tissue was removed. The molar teeth of each mandibular section were extracted, and tooth sockets were flushed. The condylar and incisal portions of the mandible were cut, and the marrow space was flushed from the inner compartments. From both the mandibular- and femur-derived bone marrow cells were positively selected using CD11b binding microbeads and magnetic column isolation (Miltenyi Biotec, catalog #130-097-142) following manufacturer’s instructions. Verification of this selection was conducted using flow cytometry (Figure S1). Briefly, flushed cells were pelleted and resuspended in Magnetic Activated Cell Sorting (MACS) buffer (phosphate-buffered saline (PBS), pH 7.2, 0.5% bovine serum albumin (BSA), 2 mM EDTA). The cells were then incubated with microbeads that bind CD11b for 15 min. The cells were then rinsed for 10 min with MACS buffer. Following incubation, the cells were applied to the MACS column in a magnetic field. The columns were rinsed 3 times. The columns were then removed from magnetic field and flushed with 5 mL of MACS buffer to elute bound CD11b+ cells. Six samples (3 femoral and 3 mandibular) were submitted for scRNA-seq. Six single-cell captures targeting 10 000 cells each were performed by ST G chip for 3′ gene expression at UMN Genomic Center.

### scRNA-seq bioinformatic analysis

Sample capture 3′ GEX library FASTQ files were processed with the 10× Genomics CellRanger Software Suite v7.0.1. CellRanger count was used to demultiplex and process cell barcodes, align reads derived from the 3′ end of transcripts to the mm10 genome, and construct a gene-by-cell unique molecular identifier counts matrix. CellRanger called between 8k and 12k cells per sample, with one sample having 23k cells per sample.

Downstream single-cell analysis was performed in R v4.1.0 using the Seurat single-cell analysis pipeline. The following quality control filtering criteria were applied to each cell from each sample: gene number between 600 and 6000 genes and mitochondrial content >20%. Filtering reduced cell numbers by about 5% per sample. Cell cycle scores for the S phase (DNA synthesis; 43 genes) and G2M phase (54 genes) were calculated per cell using the gene lists provided by Seurat.

Gene expression within each sample was normalized using SCTransform with regularization (vst.flavor = “v2”) using the “glmGamPoi” method. Mitochondrial content and differences in cell cycle score were regressed out (vars.to.regress = “percent.mt”, “CC.difference”) during normalization. Three thousand gene features were selected for finding cell-cell anchors across data sets for data set alignment or “integration” with Seurat to remove batch effects from the different captures. The uniform manifold approximation and projection (UMAP) was constructed using the top 20 principal components. Clustering was done using the Louvain algorithm with the top 20 PCs at multiple clustering resolutions. The clustree R package was constructed to visualize the clustering resolutions as a dendrogram.

SingleR, an automated cell-type classification tool, was applied using the MouseRNAData and ImmGenData from the CellDex R package.^9–11^ SingleR performs Spearman rank correlations between the single-cell transcriptome and all the samples in the bulk RNA-seq/microarray references. The single cells are annotated with the cell type labels that match the best.

Differential gene expression testing was performed on the subpopulations and 2 resolutions. The FindAllMarkers function was used to find genes that are up- and downregulated in each subpopulation versus all other cells using the Wilcoxon Rank Sum test (settings: method = wilcox, logfc.threshold = 0.3, assay = SCT, min.pct = 0.2, only.pos = FALSE). Top upregulated genes were used to help with manual cell type curation of clusters.

To find differentially expressed genes (DEGs) between femur and mandible groups for each cell type, we used the Wilcoxon rank sum test in FindMarkers with the following settings: method = wilcox, logfc.threshold = 0.25, assay = SCT, min.pct = 0.1, only.pos = FALSE. Genes were considered differentially expressed if False Discovery Rate (FDR) <0.05.

### Monocyte and osteoclast isolation

Primary BMMs were harvested from the femur and mandible of 2-mo-old male and female C57Bl/6J mice as described in the femur and mandible bone marrow section. Bone marrow monocyte selection was performed on total bone marrow. Monocytes were selected following the protocol of the mouse monocyte isolation kit (Miltenyi Biotec, catalog #130-100-629). Verification of monocyte isolation has been previously published.^6^ Briefly, flushed cells were pelleted and resuspended in MACS buffer (PBS, pH 7.2, 0.5% BSA, 2 mM EDTA). The cells were then incubated with FCR Blocking Reagent and Monocyte Biotin-Antibody Cocktail. After 5 min the cells were rinsed with MACS buffer, pelleted, and resuspended in MACS buffer. The cells were incubated with Anti-Biotin Microbeads for 10 min. After this incubation period, the cells were applied to the MACS columns in a magnetic field. Flow-through was collected as isolated monocytes. The columns were rinsed 3 times to collect unbound monocyte cells. For monocyte qRT-PCR, cells were pelleted and resuspended in TRIzol. For osteoclast differentiation, the monocyte cells were plated in 24-well plates (TPP, MIDSCI) at 2 × 10^4^ cells/cm^2^ in osteoclast media supplemented with 1.5% CMG 14-12 culture supernatant overnight. The next day, cells were re-fed with 1.5% CMG 14-12 culture supernatant and 5 ng/mL RANKL (R and D Systems, catalog #462-TEC-010) to stimulate osteoclast differentiation. Cultures were fed every other day for up to 4 d for differentiation experiments.

### RNA isolation and qRT-PCR

RNA was harvested from monocytes or osteoclasts using TRIzol Reagent (Ambion, Life Technologies, catalog #15596018) and quantified using UV spectroscopy. cDNA was then prepared from 1 μg RNA using the iScript cDNA Synthesis Kit (Bio-Rad, catalog #1708891) as per the manufacturer’s protocol. Quantitative real-time PCR was performed in duplicate using the MyiQ Single Color Real-Time PCR Detection System (Bio-Rad, catalog #172-5121). Each 20 μL reaction contained 1 μL cDNA, 10 μL iTaq Universal SYBR Green Supermix, and 500 nM forward and reverse primers. The PCR conditions were as follows: 95 °C for 3 min, and the 40 cycles of 94 °C for 15 s, 56 °C for 30 s, and 72 °C for 30 s, followed by melting curve analysis (95 °C for 5 s, 65 °C for 5 s, and then 65-95 °C with 0.5 °C increase every 5 s). Mouse primer sequences are listed in Table 1. Human primer sequences are listed in Table 2.

**Table 1:** Mouse primers used for qRT-PCR.

**Gene name**	**5′-3′ sequence**
**Ccl3 forward**	TTC TCT GTA CCA TGA CAC TCT GC
**Ccl3 reverse**	CGT GGA ATC TTC CGG CTG TAG
**Egr1 forward**	AGC GAA CAA CCC TAT GAG CAC C
**Egr1 reverse**	ATG GGA GGC AAC CGA GTC GTT
**Hprt forward**	GAG GAG TCC TGT TGA TGT TGC CAG
**Hprt reverse**	GGC TGG CCT ATA GGC TCA TAG TGC
**JunB forward**	TCA CGA CGA CTC TTA CGC AG
**JunB reverse**	CCT TGA GAC CCC GAT AGG GA
**Klf4 forward**	CGA TGA ACT GAC CAG GCA CTA
**Klf4 reverse**	CCT CTT CAT GTG TAA GGC AAG GTG
**Nr4a1 forward**	ACA GTC CTT TTG GTT CAC CC
**Nr4a1 reverse**	AAC TCC GTG GGG ATC TGT GA
**S100A8 forward**	AAA TCA CCA TGC CCT CTA CAA G
**S100A8 reverse**	CCC ACT TTT ATC ACC ATC GCA A
**S100A9 forward**	ATA CTC TAG GAA GGA AGG ACA CC
**S100A9 reverse**	TCC ATG ATG TCA TTT ATG AGG GC

**Table 2:** Human primers used for qRT-PCR.

**Gene name**	**5′-3′ sequence**
**HuCcl3 forward**	AGT TCT CTG CAT CAC TTG CTG
**HuCcl3 reverse**	CGG CTT CGC TTG GTT AGG AA
**HuEgr1 forward**	AGC AGC ACC TTC AAC CCT CAG G
**HuEgr1 reverse**	GAG TGG TTT GGC TGG GGT AAC T
**HuGapdh forward**	GAA GGT GAA GGT CGG AGT
**HuGapdh reverse**	GAA GAT GGT GAT GGG ATT TC
**HuJunB forward**	CCA CCT CCC GTT TAC ACC AA
**HuJunB reverse**	GAG GTA GCT GAT GGT GGT CG
**HuKlf4 forward**	GTC AGT CCC GGG GAT TTG TAG
**HuKlf4 reverse**	CTC CTC TGG CAT GCA GGA AC
**HuNr4a1 forward**	GGA CAA CGC TC ATG CCA GCA T
**HuNr4a1 reverse**	CCT GT TAG CCA GGC AGA TGT AC

### Immunofluorescence

Femur- or mandibular-derived BMMs were cultured in the presence of M-CSF and RANKL on coverslips until multinuclear cells appeared. Cells were fixed for 10 min at RT in 4% paraformaldehyde. Cells were permeabilized with 1% Triton X-100 for 10 min at RT. Cells were rinsed 3× with PBS. Cells were blocked for 30 min at RT in 1% BSA and 10% normal mouse serum. AMELX antibody (Abcam, catalog #153915, 1:250) was incubated overnight at 4 °C in 1% BSA. Next day, the cells were washed 3 times in PBS. Secondary antibody Brilliant Violet donkey anti-rabbit (Biolegend Catalog #406410) for 1 hr at room temperature at 1:100 dilution in blocking buffer. Cells were washed 3 times in PBS. Cells were stained with Helix NP Green (Biolegend Catalog #425303) by diluting 1:500 in blocking buffer for 15 min at RT. Antifading media was added along with coverslips. Cells were visualized on a Zeiss confocal microscope.

### Monocyte isolation and ATAC-seq

Bone marrow monocyte selection was performed on total bone marrow from femurs and mandibles as described in the above section. Collected monocyte cells were counted, and the volume required for 20 000 cells was calculated. Volumes were pelleted and washed with 50 μL of PBS. After washing, cells were pelleted and resuspended in 50 μL of ATAC-seq Lysis buffer (10 mM Tris-HCl, pH 7.4, 10 mM NaCl, 3 mM MgCl_2,_ 0.1% IGEPAL CA-630) and spun for 10 min. Cells were pelleted and resuspended in a transposition mixture consisting of 25 μL TD (Illumina catalog #121-1030), 2.5 μL TDE1 (Nextera Tn5 Transposase, Illumina Cat #FC-121-1030), and 22.5 μL nuclease-free water and incubated in mixture for 30 min. Immediately following transposition, purification was completed using a Qiagen MinElute PCR Purification Kit (catalog # 28004). Briefly, 5 volumes of buffer PB were added to 1 volume (250 μL) of PCR reaction mix. MinElute column was placed in a 2 mL collection tube. Sample was applied to the column and centrifuged for 1 min. Flow-through was discarded. The column was placed back in collection tube and centrifuged for 3 min. The column was then placed in a clean microcentrifuge tube, and DNA was eluted by adding 10 μL of Buffer EB. This was incubated for 1 min and then centrifuged for 1 min. Concentrations specifications were gathered, and samples were submitted for ATAC-seq at UMN Genomic Center.

### ATAC-seq bioinformatic analysis

Assay for transposase-accessible chromatin with sequencing, FastQC files were checked for quality with FastQC, and metrics were aggregated with multiQC. Trimmomatic was used to trim low-quality sequences and adapters. Trimmed reads were aligned to the GRCm39 *Mus musculus* genome using Bowtie2. Mapped reads with Mapping Quality (MAPQ) <20 were removed using SAMtools,^12^ and then Picard was used to mark and remove duplicates. Peak calling was performed with Genrich in ATAC mode for each pair of sample duplicates. Femur and mandible peaks were merged with bedtools merge, and then a GTF file was created using a custom Python script. Then featureCounts^13^ was used to quantify peaks for each of the 4 sample bam files and to calculate the Fraction of Reads in Peaks (FRIP) QC metric. All samples passed the ENCODE recommended FRIP score threshold of 30%, the average FRIP score was 37.9%. Differential ATAC peak analysis was conducted in R using edgeR with loess normalization and the glmQLFTest function for differential accessibility testing.^14^ Gene set enrichment analysis (GSEA) was performed using the clusterProfiler R package^15^^,^^16^ against the MSigDB Hallmark and GO Biological Processes gene sets. Peak signal visualizations were created using the rtracklayer and Gviz Bioconductor packages with normalized bigwig files created with deeptools.^17^

### Human peripheral blood mononuclear cell (PBMC) isolation and processing

#### Sample collection

This single-site, unblinded trial collected blood from 2 anatomic sites in each participant who was having third mandibular third molar(s) removed with sedation: from the arm where they had the IV started and from the third molar site(s). The surgeon was not blinded to patient identity and tracked the participants’ age, gender, specimen number, medical comorbidities, and what sedation medications were used. The basic scientists were blinded to patient identity. This study was conducted according to Good Clinical Practice (GCP) and the Helsinki Declaration. The Institutional Review Boards at the UMN approved this study, which was conducted at the UMN Oral and Maxillofacial Surgery Clinic. Recruitment of participants started in September 2023. Enrollment was completed in April 2024. Eligibility screening was performed by the surgeon on the day of the removal of third molars. The primary surgeon was a board-certified oral and maxillofacial surgeon and included in their practice surgical removal of third molars with moderate/deep sedation using the operator/anesthetist model. Sedation medications generally included midazolam, propofol, ketamine, glycopyrrolate, ketorolac, and dexamethasone. Adjunctive local anesthesia was provided, generally consisting of 2% lidocaine with 1:100 000 epinephrine and 0.5% Marcaine with 1:200 000 epinephrine. Surgeries were performed with an oral and maxillofacial surgery resident providing care under the guidance of the primary surgeon. For these surgeries, the providers performed usual care. Typically, patients consulted with the surgeon once before surgery. The surgeons reviewed the patients’ medical histories to ensure that the patients were healthy (American Society of Anesthesiologists class I or II), non-smokers, and did not have systemic disorders affecting bone. After study procedures were explained to patients, the surgeons obtained both written and verbal informed consent. After informed consent was received, the surgeon collected 2 cc of blood each from the arm of the patient during starting the intravenous (IV) via the IV catheter (20 or 22 gauge) before IV fluids were started, and from the mandibular third molar sites that had collected in these sites after the third molars were removed using an 18-gauge needle and syringe. In 78% of the extractions, bone was removed. Blood was given to the Mansky lab to perform Cd11b PBMC selection using microbeads that bind CD11b and magnetic column isolation (Miltenyi Biotec, catalog #130-097-142) and RT-qPCR to examine differences in gene expression that may exist between CD11b+ PBMCs isolated from the arm and extraction sites of the participants. Participants received Target gift cards for $10 for participation. No follow-up for study purposes was performed. Patients were followed up on to see if they had any surgical concerns.

#### Monocyte isolation and plating for KLF4 transfection

Monocytes from femur and mandible bone marrow were selected following the protocol of the mouse monocyte isolation described above. The monocyte cells were plated in 24-well plates (TPP, MIDSCI) at 20 000 cells/well in osteoclast media supplemented with 1.5% CMG 14-12 culture supernatant overnight. The following day, cells were transfected with siRNA. After 2 d, RNA was isolated from transfected cells using TRIzol Reagent and qRT-PCR was performed as described in the above section.

#### KLF4 siRNA transfection and differentiation

After isolation of monocytes, the next day, the cells were treated with transfection solution. To prepare the solution, addition of 50 μL Opti-MEM reduced serum medium phenol red-free (Gibco, catalog #11058021) was added to a sterile round-bottom tube, and 1.3 μL of 10 μM ON-TARGETplus siRNA SMARTPool was added (Horizon). In a separate round-bottom tube, add 50 μL Opti-MEM and 1.3 μL of Lipofectamine RNAi max (ThermoFisher, catalog #13778075). The 2 solutions were incubated separately for 5 min. Then, after 5 min, the DNA-Opti-MEM mixture was mixed with the Lipofectamine-Opti-MEM mixture. The solutions were incubated together for 15 min to form transfection complexes. The growth medium of the cells was replaced with 500 μL of Opti-MEM. The transfection complexes were added to the cells at a volume of 100 μL per well and allowed to incubate for 5 hr. After 5 hr, the medium was replaced with phenol-red-free alpha MEM supplemented with 10 ng/mL RANKL and 2% CMG14-12 media. Cells were harvested for RNA or tartrate-resistant acid phosphatase (TRAP) staining after 6 d of RANKL treatment when multinuclear cells had formed.

#### Tartrate-resistant acid phosphatase staining of transfected cells

Transfected cells were fixed with 4% paraformaldehyde and washed with PBS. The cells were then stained for TRAP expression using Naphthol AS-MX phosphate and Fast Violet LB salt according to protocol described in BD Biosciences Technical Bulletin #445. Cells were then imaged and photographed with light microscopy at 4× magnification, and measurements were analyzed from images (*N* ≥ 3) using NIH ImageJ software.

## Results

### Single-cell sequencing of mouse femur- and mandibular-derived CD11b+ cells reveals an inflammatory signature of mandibular-derived myeloid cells

**Figure 1:**
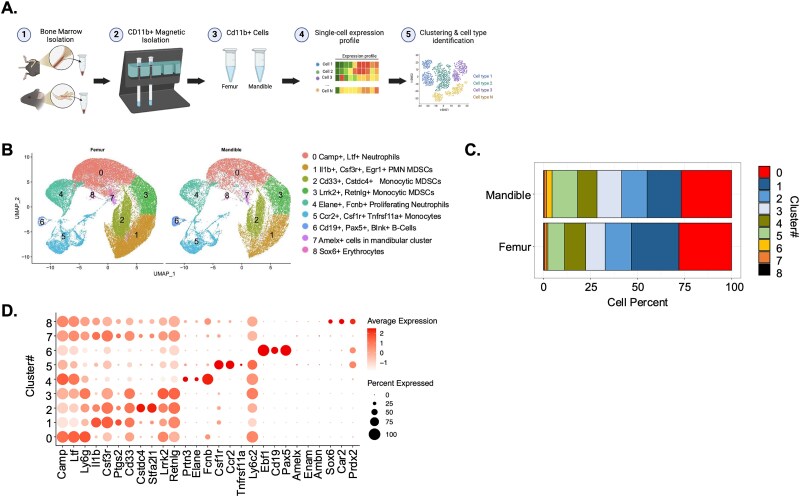
Single-cell RNA sequencing of mouse femur- and mandibular-derived CD11b+ cells. (A) Schematic of bone marrow isolation, CD11b+ cell magnetic selection, and single-cell RNA sequencing of CD11b+ cells from the femur and mandible of 2-mo-old male and female C57BL/6J mice. Six samples total were submitted for sequencing (3 femur and 3 mandible). (B) Uniform manifold approximation of sequenced cells and cluster identification by cell type. (C) Cell percentages found in each cluster. (D) expression of marker genes in each cluster.

To better understand transcriptomic differences that exist between the mandibular- and femur-derived myeloid cells, we performed scRNA-seq of CD11b+ cells. To accomplish scRNA-seq, bone marrow was flushed from both the femur and mandible, and CD11b+ cells were isolated using CD11b+ isolation beads and subsequent column magnetic isolation (Figure 1A and Figure S1). Six samples (3 femur, 2 male and 1 female, and 3 mandibles, all male) were submitted for scRNA-seq. After rigorous quality control, 5% of cells were removed from each data set. We determined mean genes per cell detected across all datasets was 2310 genes (median = 2013), and the mean unique molecular identifiers (UMIs) per cell was 10 379 (median = 7901). Nine distinct cell populations were identified using UMAP analysis (Figure 1B). We identified neutrophils, pan- and monocytic myeloid-derived suppressor cells, monocytes, and B cells based on gene expression in each cluster. Overall percentages of cells in clusters 1, 2, and 4 were increased in the femur, clusters 0 and 7 were similar between the 2 skeletal sites (Figure 1C). The percentage of cells in clusters 3, 5, 6, and 8 was increased in the mandible compared to the femur (Figure 1C). Gene expression across different clusters was used to identify the cell population found in each cluster (Figure 1D).

Cluster 0 exhibited high expression of cathelicidin antimicrobial peptide (*Camp*) and lactotransferrin (*Ltf*) genes, suggestive of neutrophilic cells (Figure 1D).^18^^,^^19^ Cluster 4 also exhibited increased expression of granulocytes/neutrophilic genes neutrophil elastase (*Elane*) and ficolin (*Fcnb*; Figure 1D and Figure S2)*.* Clusters 1, 2, and 3 contained genes expressed in polymorphonuclear and monocytic-myeloid derived suppressor cells, which are a heterogenous population of cells that expand during inflammation and regulate immune responses.^20^ These clusters include expression of genes interleukin-1beta (*Il-1β*), *CD33, stefin A2* (*Stfa2l1*), *resistin-like gamma* (*Retnlg*), *interferon-*induced *protein like 1* (*Ifitm1*), *and chemokine ligand 6* (*Ccl6*; Figure 1D and Figure S2).

Cluster 5 exhibited high expression of *Ccr2*, *Ly6c2*, and *Apoe* (Figure 1D and Figure S2). CCR2 is essential for migration of monocytes from the bone marrow to the blood during both physiological and inflammatory conditions.^21^ Monocytes express the highest APOE levels of circulating blood cells. APOE expression is inhibited by inflammatory cytokines such as IL-1β and TNF-α, and APOE binding to its receptor represses macrophage inflammatory response. Cells within cluster 5 also expressed *colony-stimulating factor 1 receptor* (*Csf1r*) and *Tnfrsf11a* (*Rank*), suggesting that these cells, if stimulated with M-CSF and RANKL, will give rise to osteoclast precursors (Figure 1D).

Divergence of lymphoid and myeloid lineage occurs before specialization of B cells; however, in cluster 6, we identified a cell population with a heightened expression of B-cell markers *Ebf1*, *Pax5*, and *Cd19* (Figure 1D). We hypothesize that these are CD11b+ pro-B cells similar to a previously described subset of B cells, which in tumor or inflammatory environments are able to transdifferentiate into macrophages.^22^

**Figure 2:**
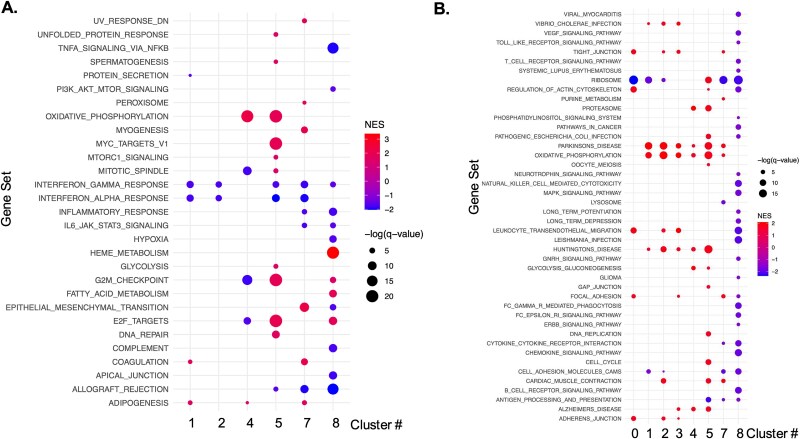
Gene set enrichment analysis (GSEA) analysis of pathways differentially regulated in mandibular-derived cell populations compared to femur-derived cell populations in each cell cluster. (A) Hallmark gene set and (B) KEGG gene set.

**Figure 3:**
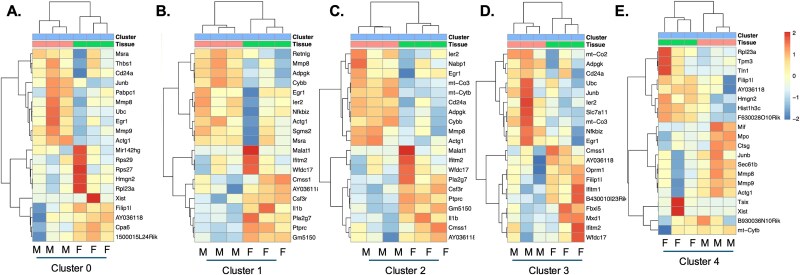
Heat maps of differentially regulated genes in clusters 0-4. (A) Cluster 0, (B) cluster 1, (C) cluster 2, (D) cluster 3, and (E) cluster 4. Abbreviations: F, femur; M, mandible.

Analyzing the changes in differentially regulated genes in each cluster using the femur-derived cells as the baseline, mandibular-derived cells had up- and down-regulated genes in every cluster (Figure S3 and Table 3). Cluster 8 had the most differentially regulated genes, followed by cluster 1, 2, and 5 (Table 3). To identify differences in gene sets or pathways enriched in each cluster based on skeletal site, we performed GSEA analysis using the Hallmark and KEGG datasets (Figure 2). Dot plots indicate gene sets or pathways that were enriched in mandibular-derived cells using femur-derived cells as the baseline (Figure 2). Mandibular-derived cells across most clusters demonstrated enrichment in mitochondrial genes involved in oxidative phosphorylation (Clusters 1, 2, 3, 4, 5, and 7; Figure 2B). Mitochondria are critical regulators of the NLRP3 inflammasome activation pathway, which senses microbial epitopes, which may result in a more inflammatory transcriptome.^23^ Besides oxidative phosphorylation genes, cell populations derived from the mandible in clusters 0 and 4 had enhanced expression of *Junb*, *Mmp8*, and *Mmp9* (Figure 3A and E). *Junb* drives neutrophil effector functions, and reductions in *Junb* expression reduced pathological inflammation in a mouse model.^24^ Femur-derived cells in clusters 1-3 had increased expression of *Il-1β*, *Ifitm2*, and *Csf3r* compared to mandibular-derived cells (Figure 3B-D). CSF3R binds to its ligand, granulocyte colony-stimulating factor (G-CSF), and activates the JAK/STAT signaling pathway, which promotes the immune suppressive functions of MDSCs.^25^ Mandibular-derived cells found in clusters 1-3 also had an increase in expression of *Nfkbiz*, a key gene involved in the regulation of NF-κB signaling pathway. Additionally, clusters 4 and 5 had enhanced proteosome gene expression, which has also been linked to increased NF-kB signaling^26^ (Figures 2 and 3B-D). Mandibular-derived cells from clusters 1-3 also had enhanced expression of metabolic genes (*Mt-Co2*, *Mt-Co3*, and *Mt-Cytb*) compared to femur-derived cells (Figure 3B-D), which supports the increase in genes involved in oxidative phosphorylation identified by GSEA.^27^ Overall, this analysis suggests a more inflammatory transcriptome in mandibular as compared to that of the femur-derived myeloid cell populations.

**Table 3:** Differentially expressed genes per cluster.

**Cluster #**	**Total number of DEGs**	**Up-regulated DEGs**	**Down-regulated DEGs**
**0**	44	15	29
**1**	214	88	126
**2**	154	61	93
**3**	50	21	29
**4**	41	10	31
**5**	143	74	69
**6**	65	3	62
**7**	88	36	52
**8**	776	256	520

Abbreviation: DEGs, differentially expressed genes.

### Identification of AMELX+ cluster

**Figure 4:**
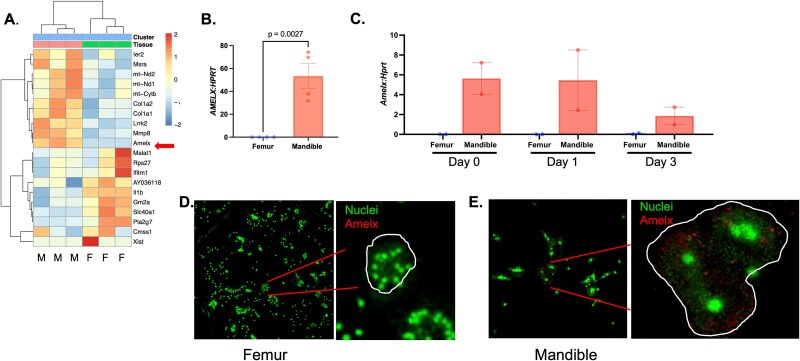
AMELX-positive monocytes and osteoclasts detected in mandibular-derived cells. (A) Heat map of differentially regulated genes in cluster 7. Red arrow indicates presence of *Amelx*. Abbreviations: F, femur; M, mandible. (B) *Amelx* expression verification in mandibular-derived monocytes by RT-qPCR. Data shown are from at least 3 independent experiments. Data is graphed relative to *Hprt*. Samples compared using a Student’s *t*-test. (C) *Amelx* expression in mandibular and femur-derived osteoclast precursors stimulated in M-CSF (day 0) or M-CSF and RANKL (days 1 and 3). (D, E) Immunofluorescence staining of AMELX+ osteoclasts at differentiation time point day 2 after RANKL addition. Data shown are from 2 independent experiments. Data is graphed relative to *Hprt.*

Cluster 7 was noted in the mandibular-derived cells as exhibiting heightened expression of *Amelx*, the gene encoding the amelogenin protein, which facilitates mineralization of the enamel matrix during tooth development (Figure 4A).^28^ First, verification of AMELX antibody staining of enamel matrix in P7 mouse tissue section was performed (Figure S4). We were also able to detect expression of *Amelx* by qRT-PCR in mandibular-derived monocytes, as well as in osteoclast precursors that were treated with M-CSF (day 0) or M-CSF and RANKL for 1 or 3 d (Figure 4B and C). There was no significant expression detected in femur-derived monocytes and osteoclast precursors (Figure 4B and C). We were also able to detect AMELX expression in mandibular-derived but not femur-derived multinuclear osteoclasts by immunofluorescence (Figure 4D and E); however, we were not able to detect AMELX+ osteoclasts in tissue sections, even though we analyzed a range of tissues from P2 through P8 day mice (data not shown). The function of this subpopulation of monocytes and osteoclasts is currently unknown; however, *amelogenin* has been reported to be expressed in cells of the hemopoietic system, but further experiments are needed to determine the role of these cells.^29^

### Validation of differentially expressed mandibular-derived monocytic genes

**Figure 5:**
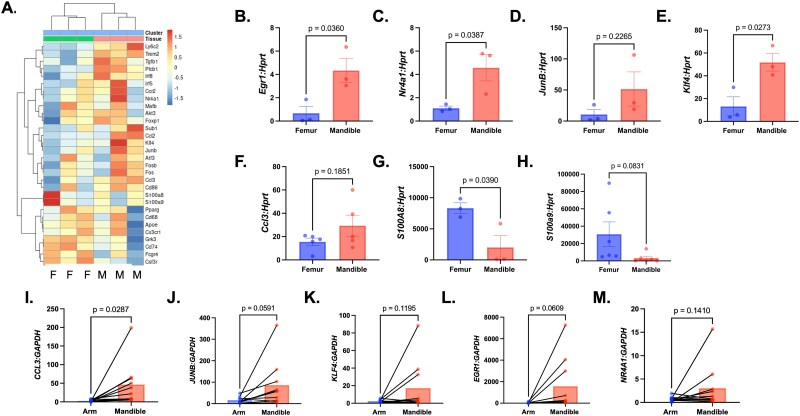
Verification of mandibular-derived differentially expressed genes in monocytes identified in cluster 5. (A) Heat-map of cluster 5 differentially regulated genes in cluster 5 in both femur and mandible. Abbreviations: F, femur; M, mandible. (B-H) Verification of upregulation of monocytic genes, including *Egr1* (B), *Nr4a1* (C), *JunB* (D), *Klf4* (E), and *Ccl3* (F), and downregulation of *S100a8* (G) and *S100a9* (H) as seen in the single-cell RNA sequencing data. Data shown are from at least 3 independent experiments. Data is graphed relative to *Hprt.* Samples were compared using Student’s *t*-test. (I-M) Genes upregulated in mouse mandibular monocytes are also upregulated in human PBMCs. Expression of human genes, including *CCL3* (I), *JUNB* (J), *KLF4* (K), *EGR1* (L), and *NR4A1* (M) was verified by RT-qPCR human CD11b+ cells isolated from PBMCs collected by IV arm line or third molar socket aspiration during third molar extraction surgery. Data shown are from 10 independent patients. Data is graphed relative to *GAPDH.* Samples were compared using Student’s *t*-test.

Given the combination of genes expressed in cluster 5, the data suggest that this cluster contains monocytes, which represent a population of cells that have the potential to differentiate into macrophages and osteoclasts; therefore, we chose to further investigate genes that were upregulated in cluster 5. Mandibular-derived cells in cluster 5 had an increase in genes involved in proliferation, as evidenced by upregulation of pathways such as E2F targets and DNA replication (Figure 2). Inflammation of tissues has been proposed to enhance proliferation as a response to repair of tissue damage.^30^ Genes shown to be upregulated in cluster 5 in the mandible-derived cells included genes such as *Nr4a1*, *JunB*, *Klf4*, and *Ccl3*, all of which are known to be involved in monocyte differentiation or proliferation (Figure 5A).^31–35^ In agreement with the previously mentioned biological pathway analysis performed on this cluster of cells, these genes have also been shown to be involved in inflammatory processes in the monocyte population. Previous evidence suggests that during inflammation, an increase in *NR4A1* expression is seen in monocyte cells. JUNB expression in monocytes is associated with pro-inflammatory cytokine secretion pathways as well as macrophage polarization. Likewise, CCL3 is a strong chemoattractant for monocytes to areas of inflammation. While EGR1 is suggested to be essential for monocyte development, much less is known about the role of KLF4 in the development of monocytes and subsequent differentiation into osteoclasts. This lack of understanding also includes how these proteins may act within the craniofacial bone.^34^ Genes shown to be downregulated in mandibular cells in the monocytic cluster included S100A8 and S100A9 (Figure 5A). Prior research demonstrated that downregulation of S100A8 and S100A9 was correlated with a decrease in prognosis in inflammatory conditions and cancers involving the oral cavity, such as head and neck squamous cell carcinoma.

Monocytes were isolated from the bone marrow of 2-mo-old C57BL/6J mice using magnetic isolation beads. Once monocytes were isolated, RT-qPCR was done to determine whether gene expression was upregulated or downregulated in the mandibular- and femur-derived cells. Genes that were significantly upregulated in the mandible-derived cells included transcription factors such as *Egr1*, *Nr4a1*, and *Klf4*, with *JunB* and *Ccl3* trending toward upregulation in the mandibular-derived samples. Both *S100a8* and *S100a9* were downregulated in the mandibular-derived monocytes (Figure 5B-H).

To explore if these trends also existed in a human model, we analyzed CD11b+ cells selected from PBMC samples collected on both male and female patients between the ages of 18 and 30 yr old who were undergoing third molar extraction surgery via IV sedation. Our data demonstrates a trend of enhanced expression in some of the abovementioned genes, including *CCL3*, *JUNB*, *KLF4*, and *NR4A1* (Figure 5I-M). While the age of all the human samples was in the same range, the variability that is noted between individual human samples may be due to differences in sedation medication, diet, physical activity, or other lifestyle-impacting factors. Together, these data demonstrate that the mandibular-derived monocytes in both the mouse model and human samples have an increasingly inflammatory transcriptomic profile as compared to the femur-derived monocytes.

### Monocytes in the mandible have increased chromatin accessibility in inflammatory pathways

**Figure 6:**
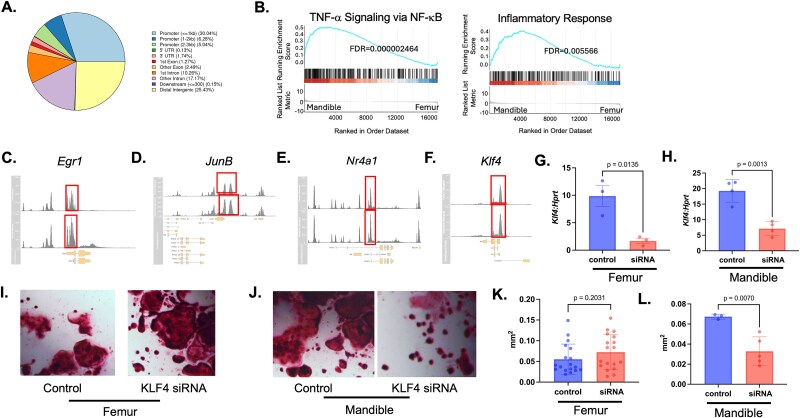
Monocytes in the mandible exhibit increased chromatin accessibility in genes associated with inflammatory processes. (A) Distribution of genomic region annotations. (B) Gene set enrichment analysis (GSEA) of mandible and femur-derived monocytes showing enrichment of genes involved in inflammatory pathways. (C-F) Assay for transposase-accessible chromatin with sequencing peak signal plots for genes *Egr1*, *Nr4a1*, *Junb*, and *Klf4*. Sequencing peak signal plot derived from femur cells is on the top and sequencing peak signal plot derived from mandible cells is on the bottom. (G-L) Monocytes were transfected with either control or *Klf4*-targeting siRNA and treated with CMG14-12 conditioned media and receptor activator of NF-kappa B ligand for 4 d. qRT-PCR of control and *Klf4* siRNA-treated (G) femur-derived monocytes (H) mandibular-derived monocytes. Representative tartrate-resistant acid phosphatase images of control and *Klf4* siRNA-treated (I) femur-derived monocytes and (J) mandibular-derived monocytes. Average size of TRAP^+^ (K) femur-derived (L) mandibular-derived cells. qRT-PCR data is graphed relative to *Hprt*. Samples were compared using Student’s *t*-test.

To further understand if the changes in gene expression, as well as the increased inflammatory signature in the mandibular-derived cells, are due to changes in chromatin accessibility, ATAC-seq was performed on monocytes collected from each of the skeletal sites. Many of the regions of open chromatin for both the femur and mandible monocytes appear at promoters (30.04%) and distal intergenic sequences (25.43%; Figure 6A). GSEA analysis was performed and showed a significant enhancement of pathways associated with “TNF-α signaling via NF-κB” (FDR = 0.000002464) and “Inflammatory Response” (FDR = 0.005566) by mandibular-derived monocytes (Figure 6B). Upon evaluation, 4 of the genes associated with cluster 5, *Egr1*, *Nr4a1*, *JunB*, and *Klf4*, observed to have increased expression by RT-qPCR in the mandibular monocytes, also exhibited increased regions of open chromatin by ATAC-seq (Figure 6C-F).

### Mandibular-derived monocytes have an increase in KLF4 expression

To better understand what transcriptomic factors may be driving the observable differences between osteoclast potential previously shown between mandibular and femoral-derived osteoclasts, we explored one of the monocyte population upregulated genes, *Krüppel-like factor 4* (*Klf4*). There is limited information known concerning the role of *Klf4* in regulating osteoclast differentiation. A previous study demonstrated that conditional loss of *Klf4* in osteoblasts results in changes in expression of RANKL and osteoclast differentiation.^36^  *Klf4* is a transcription factor involved in cellular processes such as differentiation and proliferation. To determine the necessity of *Klf4* in mandibular osteoclast differentiation, BMMs were isolated from the mandibles and femurs of 2-mo-old C57Bl/6J mice and monocytes were subsequently isolated using MACS monocyte selection beads and magnetic column isolation. We “knocked down” *Klf4* expression by siRNA transfection of femur- and mandibular-derived monocytes (Figure 6G and H). Femur- and mandibular-derived control and *Klf4* siRNA-treated monocytes were incubated with M-CSF and RANKL to induce osteoclast differentiation. Mandibular- but not femur-derived osteoclasts treated with *Klf4* siRNA were significantly reduced in size (Femur *p* = .2031 and Mandible *p* = .0070, Figure 6K and L) with the femur control cells having an average size of 0.05 mm^2^, mandibular control cells having an average size of 0.06 mm^2^, femur siRNA-treated cells having an average size of 0.07 mm^2^ and mandibular siRNA-treated cells having an average size of 0.03 mm^2^ (Figure 6I-L).

## Discussion

Several studies using advanced sequencing techniques have unveiled heterogeneity of cell function across different skeletal sites, demonstrating an accepted phenomenon.^7^^,^^8^ By exploring the differences that exist between the transcriptomic profiles of CD11b+ cells in the craniofacial region as compared to the appendicular skeleton, key upregulated genes and genetic processes in the mandibular-derived cells were revealed. Many of these changes were exhibited in genes involved in inflammatory signaling pathways. While previous literature explores differences between these 2 populations by single-cell sequencing, our study selects for a specific group, CD11b+ cells, to explore potential transcriptomic differences in several groups of myeloid precursor cells that have potential to become osteoclasts.^37^^,^^38^ A study by Jacquin et al. demonstrated that osteoclast precursors are derived from both CD117^−^ CD45^−^ CD11b^+^ and CD117^−^ CD45^−^ CD11b^−^ populations, albeit CD11b^+^ expressing cells exhibited delayed differentiation potential compared to CD11b^−^ population.^39^ A study by Jacome-Galaraza et al. indicated that there are multiple populations that may give rise to osteoclast precursors, but did not analyze the role of CD11b expression in these populations.^40^ While another study identifying osteoclast precursors in an arthritis mouse model indicated that myeloid-derived suppressor cells are the population responsible for osteoclast activity, however, it is not evident if myeloid-derived suppressor cells also form osteoclasts under physiological conditions.^41^ By focusing on the CD11b^+^ population of cells, we understand that we may have missed differences in additional cell populations that have the capability to form osteoclasts, as demonstrated by a scRNA-seq study demonstrating involvement of transient Cd11c osteoclast precursors.^42^ We focus on the population of the mandibular-derived cells in cluster 5 that expresses *Csf1r* and *Tnfrsf11a* (*Rank*) as a candidate population to explore potential mechanistic drivers of the differences observed in potential osteoclast precursors between these 2 skeletal sites, as previously described by multiple researchers.^1–3^^,^^5^

Using our single-cell data, our results revealed several avenues of future study that will allow us to continue exploring the mechanistic differences between CD11b+ cells in the craniofacial and femur bone marrow. Overall, our scRNA-seq and ATAC-seq data suggest that inflammatory pathways such as TNF-α and NF-κB signaling pathways are upregulated in mandibular-derived CD11b+ cells compared to femur-derived cells. This data is interesting when considering how the environment may affect transcriptome differences at various skeletal sites. For example, periodontal disease is a condition of the oral cavity resulting from destructive immune responses to pathogenic bacterial species. However, if inflammatory pathways are already upregulated in the cells of the bone marrow, how does an inflammatory condition such as periodontal disease affect these cells?

To our knowledge, there has been one study showing *Porphyromonas gingivalis*, a microorganism involved in periodontal disease, increases myeloid suppressor cells (MDSCs).^43^ MDSCs inhibit immune responses by T, B, and natural killer cells. MDSCs have been shown to differentiate into osteoclasts under inflammatory conditions such as rheumatoid and collagen-induced arthritis and tumor environments.^44^ Cluster 1 contains genes that have been used to identify granulocytic (polymorphonuclear, PMN) MDSCs, while monocytic (M-MDSC) genes are found in clusters 2 and 3.^7^ Future studies will involve isolating the population of cells in clusters 1-3 to determine if this population of cells contributes to osteoclast differentiation under inflammatory conditions such as periodontal disease and aging in the craniofacial bone.

Krüppel-like factor 4, a zinc-finger containing transcription factor, was shown to be upregulated in the mandible-derived cluster 5 population. KLF4 has a diverse set of functions in skeletal development, including tooth development and mineralization. In contrast, little is known about KLF4’s role in osteoclast differentiation, including osteoclasts within the craniofacial region.^45^ KLF4 is involved in regulation of key metabolic pathway participants such as MCTs.^46^ Using *Klf4* targeting siRNA, we demonstrate the necessary role of KLF4 in mandibular but not femur-derived osteoclast differentiation. In cancer cells, KLF4 has been shown to regulate expression of metabolic genes, including monocarboxylate transporters.^46^ Monocarboxylate transporters allow for the entry of monocarboxylates such as lactate into cells. Lactate can provide a unique level of epigenetic regulation through modification of histone H3 acetylation.^47^ Alternatively, lactate has been shown to modify histones through lactylation, which also modifies gene expression.^48^ Future studies need to explore the role of lactate in mandibular-derived osteoclast development. Likewise, future studies evaluating how this mechanism could serve as a potential therapeutic pathway for pathological bony conditions of the oral cavity and craniofacial region are warranted.

Cluster 6 was identified as CD11b+ B cells. These cells have been described in a cancer model to be able to transdifferentiate into tumor-promoting macrophages.^22^ The CD11b+ cells are reported to respond to the M-CSF produced by tumor cells and downregulate the expression of *Pax5*, allowing them to transdifferentiate into macrophages.^22^ Through single-cell and bulk RNA sequencing, B cell-derived macrophages are functionally distinct from monocyte-derived macrophages.^22^ The function of these B cells in the craniofacial region is not currently known, but in a study of humans with severe periodontal disease, these cells disappeared compared to healthy controls.^49^ Cluster 7 contained *Amelx*+ cells, which were detectable in mandibular-derived monocytes and osteoclasts (day 0-3) but were undetectable in femur-derived cells. We only tested 2 biologically independent samples of osteoclasts for *Amelx* expression, so we are unable to conclude if there are significant differences in *Amelx* expression at distinct times during differentiation of mandibular-derived osteoclasts. The functions of these AMELX+ cells within craniofacial bone are not currently known, but it is speculated that they might have a role in development and stress-induced bone remodeling.^50^

In clusters 1, 2, and 5, we identified increases in a glucose transporter, monocarboxylate transporters, and mitochondrial genes. Recent evidence demonstrates that cell metabolism and inflammation are linked. Under inflammatory conditions, immune cells have a need to generate energy to support cellular growth and expression of proinflammatory cytokines. Metabolites are also able to regulate gene expression through changes in acetylation or α-ketoglutarate as a substrate for histone demethylases. Future studies will determine how changes in cell metabolism allow cells in the mandible to respond to the changing craniofacial environment.

Overall, our study indicates that myeloid cell populations in the mandible have a more inflammatory gene signature, suggesting that the skeletal location influences gene expression. We determined that mandibular monocytes have increased areas of open chromatin in gene pathways involved in inflammation and TNF-α signaling via NF-κB. These open regions contained genes such as *Egr1*, *Nr4a1*, and *Klf4*, which were also upregulated in our monocyte cluster. Loss of *Klf4* expression by siRNA in mandibular- but not femur-derived monocytes led to a decrease in osteoclast differentiation, suggesting that loss of certain genes in monocytes may have a more profound effect depending on the skeletal location.

## Supplementary Material

Sup_Figure_1_ziaf074

Sup_Figure_2_ziaf074

Sup_Figure_3_ziaf074

Sup_Figure_4_ziaf074

## Data Availability

The data discussed in this publication have been deposited in NCBI’s Gene Expression Omnibus and are accessible through GEO Series accession number GSE285313 (https://www.ncbi.nlm.nih.gov/geo/query/acc.cgi?acc=GSE285313) and GSE285314 (https://www.ncbi.nlm.nih.gov/geo/query/acc.cgi?acc=GSE285314).
