# Supplementary figures and images for: Skeletal site-specific variations in myeloid cells: insights from single-cell RNA sequencing of the mandible and femur
Source: JBMR Plus. 2025 Apr 24;9(7):ziaf074. doi: 10.1093/jbmrpl/ziaf074 (PMC12143482; doi:10.1093/jbmrpl/ziaf074)

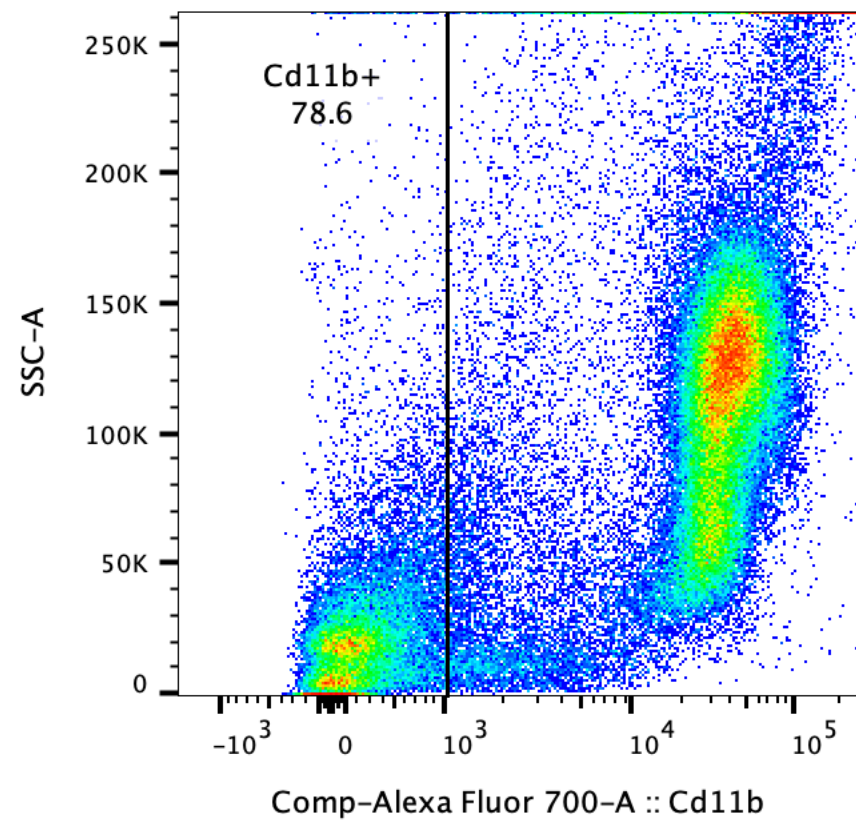

Supplement: Sup_Figure_1_ziaf074 [file sup_figure_1_ziaf074.pdf]

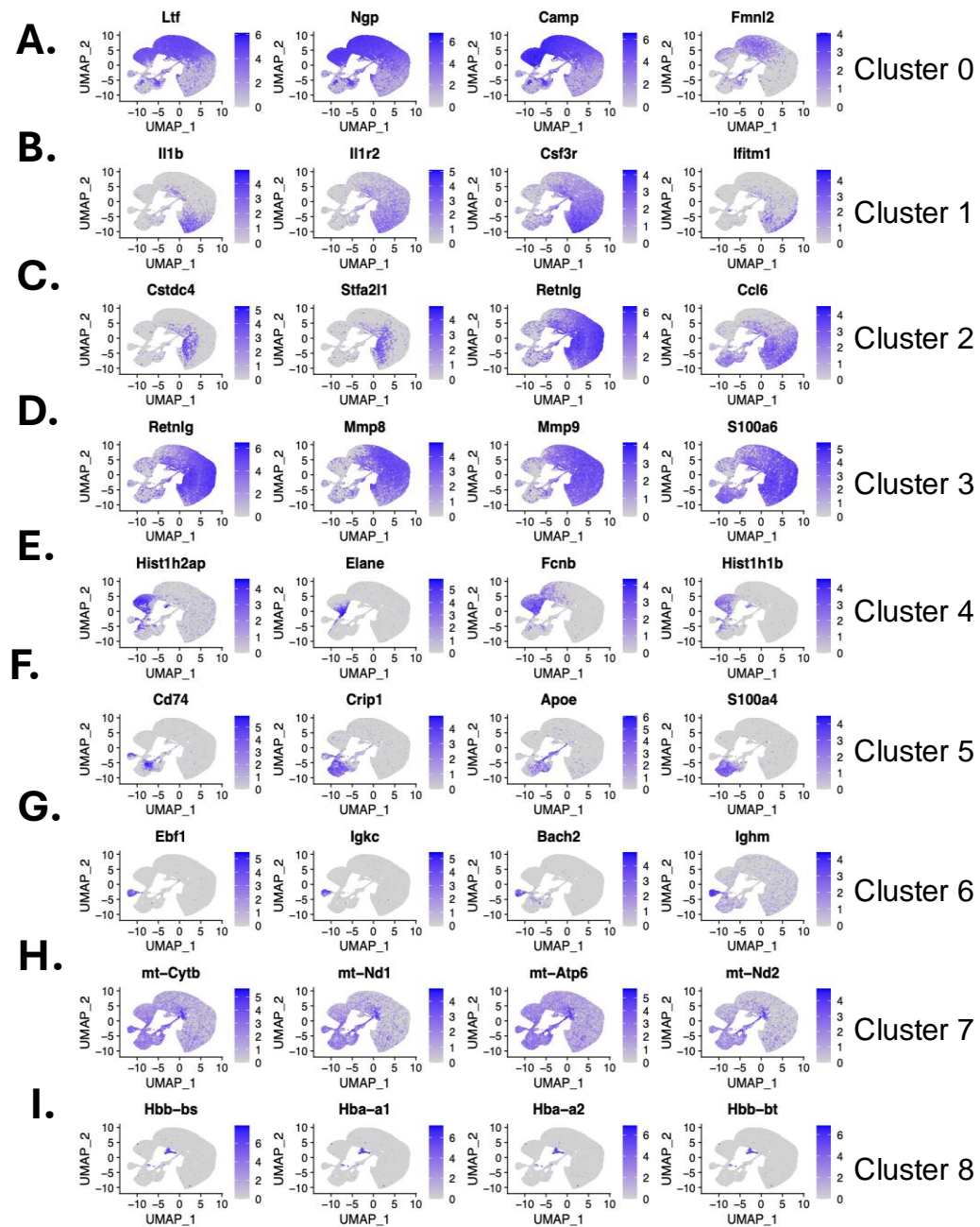

Supplement: Sup_Figure_2_ziaf074 [file sup_figure_2_ziaf074.pdf]

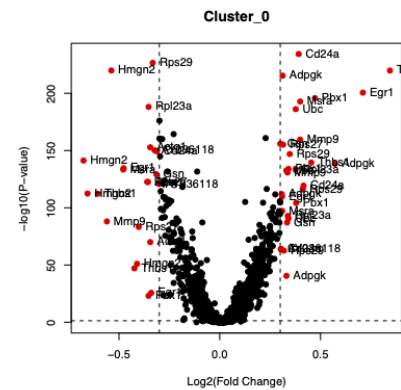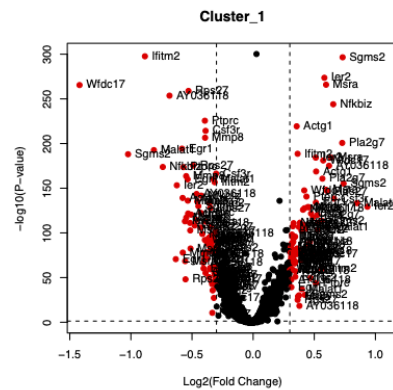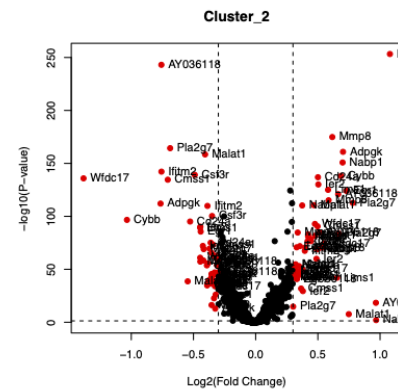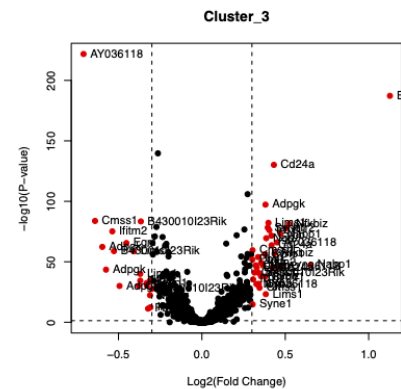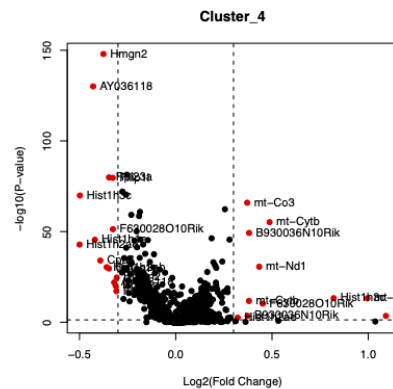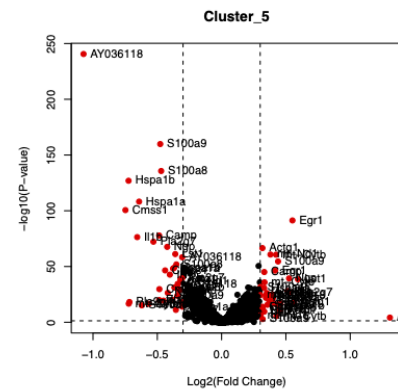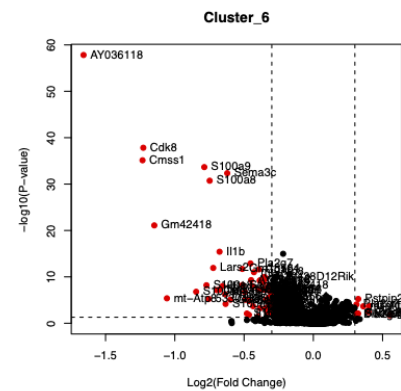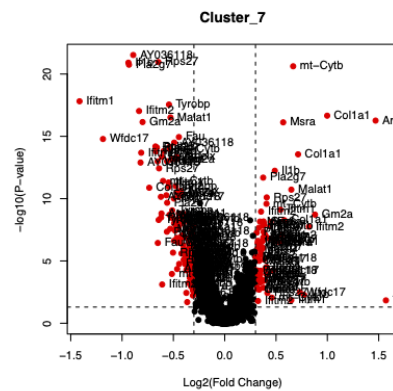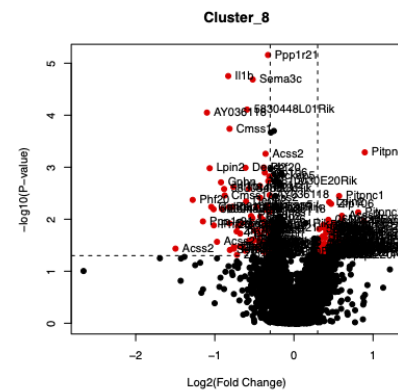

Supplement: Sup_Figure_3_ziaf074 [file sup_figure_3_ziaf074.pdf]

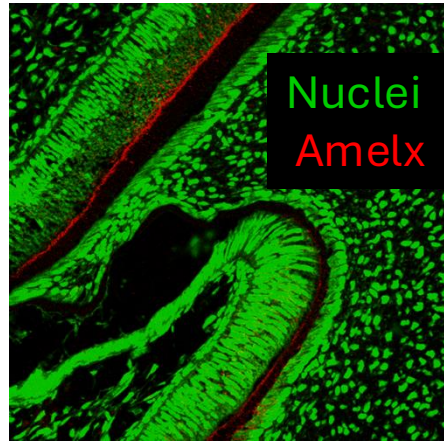

Supplement: Sup_Figure_4_ziaf074 [file sup_figure_4_ziaf074.pdf]
